# A global dataset on subsidies to the fisheries sector

**DOI:** 10.1016/j.dib.2019.104706

**Published:** 2019-10-23

**Authors:** U. Rashid Sumaila, Daniel Skerritt, Anna Schuhbauer, Naazia Ebrahim, Yang Li, Hong Sik Kim, Tabitha Grace Mallory, Vicky W.L. Lam, Daniel Pauly

**Affiliations:** a Institute for the Oceans and Fisheries, University of British Columbia, 2202 Main Mall, Vancouver, BC, V6T 1Z4, Canada; b School of Public Policy and Global Affairs, University of British Columbia, 2202 Main Mall, Vancouver, BC, V6T 1Z4, Canada; c Ocean University, China; d China Ocean Institute and University of Washington, United States

**Keywords:** World Trade Organization, Fisheries, Subsidies, Capacity-enhancing, Overcapacity, Overfishing, Fuel subsidies, Fisheries management

## Abstract

This article contains data on subsidies provided to the fisheries sector by maritime countries. The dataset is the culmination of extensive data collection efforts using peer-reviewed and grey literature, national budgets, online databases, websites and other relevant sources (e.g. OECD, World Bank and WTO), in order to estimate the scope and magnitude of global fisheries subsidies. For subsidies where we found evidence of expenditure by a country, we record the total amount alongside the source references and refer to these as ‘reported’ data. Where evidence is found that a country provides a subsidy but no amount reported, we estimate using various approaches and refer to these as ‘modeled’ data. Where evidence exists that no subsidy is provided by a country we refer to these null values as ‘not found evidence of subsidy’. All amounts were converted to constant 2018 USD using 2017 exchange rates and annual Consumer Price Index averages. The final dataset of ‘reported’, ‘modeled’ and ‘not found’ subsidies for 2018 consists of 13 subsidy types across 152 maritime countries. The dataset, first developed in the early 2000s, now forms part of the global fisheries management infrastructure and is a central tool used by WTO negotiators. The data we provide may be used to support local, regional and global fisheries management decision-making and may have further uses when analysed in combination with other fisheries related data. Interpretation of these data can be found in the associated research article titled “*Updated estimates and analysis of global fisheries subsidies*” [1].

**Table:** Specifications Table

Subject	Management, Monitoring, Policy and Law
Specific subject area	Oceans and fisheries economics
Type of data	Table
How data were acquired	Raw data acquired from 165 individual publicly accessible data sources.
Data format	Raw and Analysed
Parameters for data collection	Data were collected for 33 different fisheries subsidies sub-types and aggregated to 13 fisheries subsidy types per maritime country. Most recent data available were retrieved.
Description of data collection	Data were collected from a number of major sources including; federal and state budgets; WTO Subsidies and Policy Notifications; the OECD's Fisheries Support Estimates; national fisheries department reports and financial summaries; European Commission Operational Plans and Annual Implementation Reports for the EMFF; peer-reviewed and grey literature; personal communication with academics and country officials; national financial law documents; and national tax expenditure reports. All raw data on subsidy amounts were stored with the relevant data sources, and subsequently aggregated to form the final subsidy dataset alongside ‘modeled’ values. ‘Modeled’ values were produced using a series of secondary data sources and the raw data in order to provide estimates of fisheries subsidies by type for countries where no quantitative data was available, but evidence was found that the subsidy exists.
Data source location	Data include subsidy values for 152 maritime countries.
Data accessibility	Data are available with this article.
Related research article	U. Rashid Sumaila, Naazia Ebrahim, Anna Schuhbauer, Daniel Skerritt, Yang Li, Hong Sik Kim, Tabitha Grace Mallory, Vicky W.L. Lam, Daniel Pauly. Updated estimates and analysis of global fisheries subsidies. Marine Policy (2019) [1]

**Table:** 

**Value of the Data** • Mounting evidence of the negative impacts of the provision of certain fisheries subsidies has led to worldwide commitments to effectively limit and manage their use. However, data on the scope and magnitude of fisheries subsidies are often unreliable or not available. This dataset helps to address this and as such supports global fisheries subsidies discussions and decision-making. • The data can be used by scientists, non-governmental organisations, policy-makers, and government agencies and ministers, to inform research and decision-making regarding the subsidisation of the fisheries sector. • The data can be used to derive insight and create policies for improving country-level or regional fisheries management. The data can also be used for informing discussions and negotiations of global fisheries subsidies reform. The dataset may have further uses when analysed in combination with other fisheries datasets. • The raw data and secondary data to construct the dataset come from publicly available sources and are all included within this article.

## 1 Data

The dataset in this article describes the subsidies estimated to be provided to the fisheries sector in 2018 by 152 maritime countries across 13 identified subsidy types (see; Sumaila_dataset.csv). The dataset was constructed using a number of secondary data sources and raw data on subsidy amounts.

Sumaila_rawdata.csv describes the raw subsidies data on the amount of fisheries subsidies at the subsidy sub-type level per maritime country. These raw data were retrieved from 165 individual publicly accessible data sources and covers the time period 2009 to 2019. Sumaila_unkown.csv describes all the sources of evidence for ‘unknown’ values, that is where the presence of subsidy was found, but with no amount. Sumaila_modelcode.pdf provides a single model script that utilises all the raw data, unknown data, and secondary data sources to generate the final dataset.

## 2 Experimental design, materials, and methods

Our methodology to generate the global fisheries subsidies dataset centres on the compilation of raw data within a database organised by maritime country (*n* = 152) and by subsidy type (*n* = 13). Our approach to estimating total expenditure for each subsidy type per country (*n* = 1976) consisted of the following broad steps:

• Evidence was gathered on whether a given type of fisheries subsidy, *j*, is provided by a given country, *i.* Note that in the case of the subsidy type ‘fisheries management’, we assume that all maritime countries with fishing fleets spend public funds to manage their fisheries;
• For subsidy types for which we find raw data of expenditure, we record the amount reported with the relevant source reference. Multiple raw data for a single subsidy type in the same year are totalled to provide a single amount in the dataset. We refer to these amounts as ‘reported’ data in the dataset;
• If evidence is found that a country provides a subsidy but no amount is reported in available sources we fill the missing numbers using the estimation approaches described below. We refer to these amounts as ‘modeled’ data in the dataset. We provide a list of all the ‘unknown’ values with this article, i.e. a list of sources of evidence of the presence of subsidy, but with no amount (see; Sumaila_unkown.csv);
• If no evidence is found of the presence of a subsidy, we then search reported data in Ref. [2], assuming that subsidies reported therein continue to exist today, and again fill the missing numbers with ‘modeled’ data using the estimation approaches below; and
• If explicit evidence is found to the contrary, i.e. that a subsidy is not provided by a country, or no evidence is found and no value was reported within [2], we then enter a null value and refer to these as ‘not found evidence of subsidy’ in the dataset.

### 2.1 Subsidy categorisation

The subsidy categorisation applied here is based on the subsidy's possible impact on natural resources over time and is founded upon economic theory. All direct and indirect transfers from the public sector to the private sector (here the fishing sector) are included. We use three broad subsidy categories; beneficial, capacity-enhancing and ambiguous. Each category is further divided, giving a total of 13 subsidy types: 1) Beneficial: fisheries management; fisheries research and development; and marine protected areas; 2) Capacity-enhancing: boat construction, renovation and modernisation; fisheries development programs; fishing port development; marketing and storage infrastructure; tax exemptions; fuel subsidies; and fishing access agreements; and 3) Ambiguous: fisher assistance; vessel buyback; and rural fisher community development programs. For the purpose of recording raw data, subsidy types are further divided into 33 subtypes (see; Sumaila_rawdata.csv). These sub-types provide the framework of our subsidies categorisation and are used as a guideline to decide which kind of reporting financing would fall into which subsidy type and category. However, we do not analyse the data at this level.

### 2.2 Raw data collection

The most recent data and information available on marine fisheries subsidies were retrieved from the following major sources: a) federal and state budgets; b) WTO subsidies and policy notifications; c) the OECD's Fisheries Support Estimates; d) national fisheries department reports and financial summaries; e) European Commission annual implementation reports for the EMFF and Operational Programmes f) peer-reviewed and grey literature; g) personal communication with academics and country officials; h) national financial law documents; and i) national tax expenditure reports. All the information on sources per country and subsidy sub-type are provided within the attached raw data file (see; Sumaila_rawdata.csv).

We included information on subsidies from the years 2009–2019. When a reported amount was retrieved, the following decisions were made: 1) Which subsidy sub-type best describes the reported amount based on the available description in the original data source; 2) If the reported amount covered multiple sectors in addition to marine capture fisheries, such as for fisheries and aquaculture for example, the amount was split accordingly while assuming equal distribution between the reported sectors, unless otherwise specified; and 3) If a reported total amount was for a programme that spans more than one year, we assume equal distribution between each year and report the total amount divided by the give numbers of years, unless the division is provided. Raw data are recorded in the local currency and alongside the date and source details. All retrieved amounts were converted from local currency into USD using 2017 exchange rates from The Bank for International Settlements [3], the most complete and recent source found, and all currencies were reported as currency codes in the raw data (see; Sumaila_rawdata.csv). Using annual averages of Consumer Price Index (CPI) data from the International Monetary Fund for USA [4], we then converted all numbers to constant 2018 USD. The sum of all raw data for each subsidy type per maritime country in constant 2018 USD were then included in the subsidies dataset (see; Sumaila_dataset.csv).

### 2.3 Estimating missing subsidy values

Where evidence existed, either from Ref. [2] or from other sources (see; Sumaila_unkown.csv), that a subsidy type was provided by a country but no amounts were available, we used a number of approaches to model the amount. Depending on the subsidy type, we used four different approaches to estimate missing subsidies: a general approach for filling gaps for all subsidy types, except in the case of fuel subsidies, fishing access agreements, and marine protected areas (MPAs), for which we used bespoke approaches. All the above approaches are included within a single model script attached to this article (see; Sumaila_modelcode.pdf), and to ensure replicability the secondary data sources that are used in combination with the raw data to model missing subsidies are referenced and include the following:

- **Sumaila *et al.* (2016)**; used to identify where to fill missing subsidies with ‘modeled’ data [2];
- **Bank for International Settlements**; exchange rates used to convert currencies to USD [3];
- **International Monetary Fund for USA**; annual average Consumer Price Index used to convert all amounts to constant 2018 USD [4];
- **Sea Around Us**; total landed value per country for landings taken from within the country's EEZ and for landings by the country's fishing fleet, were used to calculate subsidy intensity values and to estimate fees paid for fishing access to other countries EEZs [5];
- **United Nation Human Development Index**; 2017 data used to classify countries in to two country classes [6];
- **Greer *et al.* (2019)**; reported fisheries fuel consumption by country used to calculate subsidy per tonne of fuel [7];
- **Belhabib *et al.* (2015)**; reported ratios of compensation paid for access and landed value from other countries EEZs used to estimate fees paid for fishing access to other countries EEZs [8];
- **The World Database on Protected Areas**; data on protected areas in each country used to estimate subsidies on MPAs [9]; and
- **McCrea-Strub *et al.* (2011)**; estimated MPA maintenance costs and estimated MPA establishment costs used to estimate subsidies on MPAs [10].

### 2.4 General approach

First, we calculated subsidy intensity (*SI*), defined as the ratio of subsidy amount and total landed value (*LV*), using reported data for each subsidy type per country. LV information was taken from 2010 *Sea Around Us* data per country [5]. This data source provides reconstructed catch estimates that consider the contribution of illegal, unreported and unregulated fish landings. Two estimates of LV are provided per country for landings taken from within the country's exclusive economic zone (EEZ) and for landings by the country's fishing fleet. The SI for each subsidy type was therefore calculated using the most appropriate LV estimate, based on whether the subsidy is more likely to impact the country's fishing fleet or fishing within the country's EEZ. Subsidy types for fisheries management, research and development, and rural fisher development were grouped into the EEZ and subsidy types for boat construction, port construction, fisheries development, marketing infrastructure, buyback programs, fisher assistance programs, and tax exemptions, were assigned to the country's fishing fleet group.

Following previous methodologies [e.g. 1,11], we assume that subsidy payments vary depending on the economic development of a country, and therefore our approach considers two country groups separately. We used the 2017 United Nation Human Development Index (HDI) as an indicator of development status [6]. We subsequently grouped all countries into two groups, using UNDP's cut-off point of HDI less than 0.7 for low/medium HDI countries, and above or equal to 0.7 for high/very high HDI countries. This means that we have combined the low/medium (below HDI 0.7, n = 58) and high/very high (above or equal HDI 0.7, n = 94) groups of countries, that we refer to as ‘low’ and ‘high’, respectively. We considered this approach to provide better robustness and accuracy for estimating missing subsidy values as explained in the following steps.

The mean *SI* per subsidy type (*SI*_*j*_) for each HDI group was then multiplied with each country's landed value to estimate missing amounts for data points that we found evidence that the subsidy is being provided, but without quantitative data (Eq. (1)).

(1) $$\;Subsidy_{i,j}=SI_{j}\cdot LV_{i}$$

where, *Subsidy*_*i,j*_, is the unknown amount for subsidy *j* for country *i*, *SI*_*j*_ is the mean subsidy intensity across all known data points for subsidy *j* within the same HDI group as country *i*, and *LV*_*i*_ is the landed value for country *i*.

### 2.5 Fuel subsidies

This method is based on Sumaila et al. (2008) [12]. For the current estimate of missing data, we used the reported data we collected and information for fisheries fuel consumption reported in Greer (2019) [7]. We calculated subsidy per tonne of fuel used and the mean of reported fuel subsidies for each HDI group. Missing values were computed by multiplying each country's fuel consumption by the mean subsidy per tonne of fuel for the relevant HDI group. For countries where no information was found, instead of assuming that zero subsidies were provided, we used information from previous studies [2] as an indication whether fuel subsidies were provided then, and if yes, we applied our method to estimate the missing values.

### 2.6 Fishing access agreements

Sea Around Us data [5] on landed value (USD) by location for each country was used to estimate fees paid for fishing access to other countries EEZs. To determine how much of a country's total landed value is taken from the host EEZ, we deduct from its total landed value the proportion caught in its own EEZ (including overseas territories and dependencies) and the high seas, and for fish caught by EU Member States from other Member State EEZs.

Belhabib et al. (2015) estimated a ratio of compensation paid by for access to landed value from other countries EEZs [8], including those for illegally caught fish. The authors estimated that, on average, ratios were 8% and 4%, respectively for the compensation paid by the EU and China to access West African waters. Based on this, we assumed a compensation rate of 6% of adjusted mean LV from 2005 to 2014 and use this to estimate subsidies for access to other country EEZs. This approach makes a crucial assumption that countries are indeed paying for the privilege of access to other countries fish. Due to the dearth of information regarding fees paid by public entities for access to fish, this approach was considered the most likely to ensure that we captured all possible contributions for access, whether direct financial contributions or other. It is also important to note that these estimates do not include fees received by a country for access to their resources, but the outgoing expenditure of the fishing country.

### 2.7 Marine protected areas

The subsidies spent on MPAs in a given year by a country is equal to the cost of the new MPAs established that year (establishment cost, EC) plus the cost of running all existent MPAs in the country that year (maintenance cost, MC). Based on the literature, we determine the per unit area (km^2^) cost of establishing and running an MPA in a given country [10,13]. The total expenditure on MPAs by a country in a given year (TE) is expressed in Equation (2).

(2) TE=EC+MC

where *EC* = *xAME* denotes total establishment cost of MPAs; *x* is the cost per unit area of MPA established, and *AME* represents area of MPA established. *MC* = *yAMPA* is the total running cost of MPAs; *y* is the cost per unit area of MPAs being run and maintained, and *AMPA* is the existing MPA area in a country.

The World Database on Protected Areas (WDPA) provided data on protected areas in each country [9], including; size of individual protected areas, size of marine area protected, the year of establishment and the country where the protected area is located. From these *AME* and *AMPA* were calculated for each country. As the costs of running and establishing an MPA have been shown to increase nonlinearly with increasing MPA size [10], protected marine area was summed across eight size categories. Total area of protected marine space for each size category within each country was then multiplied by estimated maintenance costs, and the total area of protected marine space established since 2018 for each of the eight size categories was multiplied by estimated establishment costs, both as reported by McCrea-Strub et al. (2011) [10]. This approach makes a crucial assumption that reported protected area coverage is being implemented and enforced sufficiently, both in order to accrue costs and to be effective at enhancing fisheries either directly or indirectly. This method makes the assumption that each country that has one or more MPA, spends money on it some way.

### 2.8 Global subsidies dataset

The model code provided (see; Sumaila_modelcode.pdf), along with the raw data (see; Sumaila_rawdata.csv), unknown subsidy amounts (see; Sumaila_unknown.csv) and secondary data sources, allow the production of the final global subsidies dataset (see; Sumaila_dataset.csv). All values are converted to 2018 constant USD and thus represent to the extent possible all fisheries subsidies provided in 2018 by 152 maritime countries for the 13 subsidy types identified.

## References

[1] Sumaila U.R., Ebrahim N., Schuhbauer A., Skerritt D., Li Y., Kim H.S., Mallory T.G., Lam V.W.L., Pauly D. (2019). Updated estimates and analysis of global fisheries subsidies. Mar. Policy.

[2] Sumaila U.R., Lam V., Le Manach F., Swartz W., Pauly D. (2016). Global fisheries subsidies: an updated estimate. Mar. Policy.

[3] Bank for International Settlements (2019). Effective exchange rate indices for 2017. https://www.bis.org/statistics/eer.htm.

[4] International Monetary Fund for USA (2019). Country indexes and weight. https://data.imf.org/regular.aspx?key=61015892.

[5] Pauly D., Zeller D. (2015). Sea Around Us Concepts, Design and Data. http://www.seaaroundus.org/data/#/eez.

[6] United Nations Development Programme (2019). Human development index and its components. http://hdr.undp.org/en/composite/HDI.

[7] Greer K., Zeller D., Woroniak J., Coulter A., Winchester M., Palomares M.D., Pauly D. (2019). Global trends in carbon dioxide (CO2) emissions from fuel combustion in marine fisheries from 1950 to 2016. Mar. Policy 107.

[8] Belhabib D., Sumaila U.R., Lam V.W., Zeller D., Le Billon P., Kane E.A., Pauly D. (2015). Euros vs. Yuan: comparing European and Chinese fishing access in West Africa. PLoS One.

[9] U.-W. IUCN (2010). The World Database on Protected Areas (WDPA), Annual release.

[10] McCrea-Strub A., Zeller D., Sumaila U.R., Nelson J., Balmford A., Pauly D. (2011). Understanding the cost of establishing marine protected areas. Mar. Policy.

[11] Sumaila U.R., Khan A.S., Dyck A.J., Watson R., Munro G., Tydemers P., Pauly D. (2010). A bottom-up re-estimation of global fisheries subsidies. J. Bioecon..

[12] Sumaila U.R., Teh L., Watson R., Tyedmers P., Pauly D. (2008). Fuel Price increase, subsidies, overcapacity, and resource sustainability. ICES J. Mar. Sci..

[13] Balmford A., Gravestock P., Hockley N., McClean C.J., Roberts C.M. (2004). The worldwide costs of marine protected areas. Proc. Natl. Acad. Sci..

