# Supplementary material for: A global dataset on subsidies to the fisheries sector
Source: Data Brief. 2019 Oct 23;27:104706. doi: 10.1016/j.dib.2019.104706 (PMC6920478; doi:10.1016/j.dib.2019.104706)
Supplement: Multimedia component 1 [file mmc1.zip › Suppl Mat/Sumaila_modelcode.pdf]

In [2]:
```
%matplotlib inline
import pandas as pd
import matplotlib.pyplot as plt
import numpy as np
plt.style.use('ggplot')
pd.__version__
```

Out[2]: '0.23.4'

In [3]:
```
pwd
```

Out[3]: '/Users/annaschuhbauer/Python/Subsidies_2019/Final_2018'

In [4]:
```
Subsidies2009 = pd.read_csv("Input/Subsidies_2009.csv")
# original file: Sumaila et al. 2016
Countrydata = pd.read_csv("Input/Country_Data.csv")
# compiled from various sources online
Subsidytypes = pd.read_csv("Input/Subsidy_types.csv")
# includes Fisheries Subsidies categories and types
Subsidies2019 = pd.read_csv("Input/Subsidies2019.csv")
# raw data by subsidy subtype
CPI =  pd.read_csv("Input/CPI_IMF.csv")
HDI = pd.read_csv("Input/HDI_2017.csv")
Currency = pd.read_csv("Input/Currency_2017.csv")
Country_status_main = pd.read_csv("Input/Country_Status_Main.csv")
Countries_class = pd.read_csv("Input/Countries_class.csv")
EEZ_LV = pd.read_csv("Input/EEZLV_2014.csv")
Fleet_LV = pd.read_csv("Input/FleetLV_2014.csv")
Type_Fleet_EEZ = pd.read_csv("Input/Subtype_Fleet_EEZ.csv")
Fuel_consumption = pd.read_csv("Input/Fuel_consumption.csv")
Unknown = pd.read_csv("Input/Unknown_amounts.csv")
# all files in csv format from airtable (Subsidies2019, Unknown), from S
ea Around Us (EEZ_LV and Fleet_LV)
# 2009 subsidies data, downloaded from internet (CPI, HDI, Countrydata,
 Currency) and created (Type_FLeet_EEZ)
```

In [5]:
```
Countries = Countrydata.loc[:,['Country','Subregion']]
# reduce dataframe to only necessary columns
```

In [6]:
```
HDI_merged = HDI.replace('VERY_HIGH', 'HIGH')
HDI = HDI_merged.replace('MEDIUM', 'LOW')
# use two main groups ('Very High and High' = High and 'Medium and Low'
 = Low) based on UN information
```

In [7]:
```
Countries_HDI = pd.merge(left=Countries, right=HDI, how='left')
# merge country data with HDI groups
```

In [8]:
```
Subsidies_currency = pd.merge(left=Subsidies2019, right=Currency, how='l
eft')
# merge currency with subsidy data to prepare for conversion into USD
```

In [9]:
```
Subsidies_currency['USD 2017 exchange rate'] = Subsidies_currency['Amoun
t']*Subsidies_currency['USD2017']
# create a new column showing all subsidy amounts converted into USD usi
ng 2017 exchange rate data
```

In [10]:
```
CPI2018 = CPI['CPI'][63]
CPI2018
# define 2018 CPI value to use in further calculating 2018 real constant
USD values for all Subsidy Subtypes
```

Out[10]: 115.15730320000002

In [11]:
```
Subsidies_CPI = pd.merge(left=Subsidies_currency, right=CPI, how='left')
# merge Subsidies data by subtype level with the anual CPI per year
```

In [12]:
```
Subsidies_CPI['Subsidies Constant 2018 USD'] = Subsidies_CPI['USD 2017 e
xchange rate'] * CPI2018/Subsidies_CPI['CPI']
# formula CPI adjusted value to Subsidies in Constant 2018 USD:
# new 2018 value = original estimate * CPI2018/original year CPI
# original value if from 2015 = 1000 -- > X = 1000 * 115/89 = XX
```

In [13]:
```
Subsidies_2018USD = Subsidies_CPI[['Country','Type','Subtype','Class','S
ubsidies Constant 2018 USD']]
# reduce dataframe to only necessary columns to prepare for grouping
```

In [14]:
```
Subsidies_2018_Types = Subsidies_2018USD.groupby(['Country','Class'], as
_index=False).sum()
# group subsidy subtypes(n=33) into subsidy Types (n=13)
# Subsidy Types are here identified as Class (from A1 - C3)
# now all amounts in Constant 2018 values by Subsidy Type
```

In [15]:
```
Subsidies_2018_HDI = pd.merge(left=Subsidies_2018_Types, right=Countries
_HDI, how='left')
# merge with HDI data to compute averages per HDI group which will be us
ed to fill gaps
```

In [16]:
```
HDI_LV_E = pd.merge(left=Subsidies_2018_HDI, right=EEZ_LV, how='left')
# merge Landed Value data per country EEZ with Subsidies and HDI datafra
me
HDI_LV = pd.merge(left=HDI_LV_E, right=Fleet_LV, how='left')
# merge Landed Value data per country fishing fleet with above subsdidy
 HDI and L_EEZ data
```

In [17]:
```
Type_Fleet_EEZ = Type_Fleet_EEZ[['Class','LV_Type']]
# shorten EEZ and Fleet dataframe to use to identify if subsidy intensit
y is calculated by fleet
# or EEZ depending on subsidy Type (Class)
```

In [18]:
```
HDI_LV_short = HDI_LV[['Country','Class', 'Subsidies Constant 2018 USD',
                        'HDI_group', 'FleetLV_USD_2014', 'EEZLV_2014_USD'
]]
# reduce dataframe to only necessary information
```

In [19]:
```
HDI_LV_short = pd.merge(left = HDI_LV_short, right = Type_Fleet_EEZ, how
= 'left')
# merge subsidies, landed value, HDI group information with fleet or EEZ
allocation per subsidy Type (Class)
```

In [20]:
```
HDI_LV_short['Subsidy_intensity'] = np.where(HDI_LV_short['LV_Type']=='E
EZ',
                                             HDI_LV_short['Subsidies Con
stant 2018 USD']/
                                             HDI_LV_short['EEZLV_2014_US
D'],
                                             HDI_LV_short['Subsidies Con
stant 2018 USD']/
                                             HDI_LV_short['FleetLV_USD_2
014'])

# compute subsidy intensity by dividing subsidies over landed value
# use landed value from Country EEZ or Fleet depending on subsidy type i
ndicating EEZ or FLeet
```

In [21]:
```
Subsidies_HDI_SI = HDI_LV_short[['Country', 'Class','HDI_group', 'Subsid
y_intensity']]
Subsidies_SI = Subsidies_HDI_SI.groupby(['HDI_group','Class'], as_index=
False).mean()
Subsidies_SI_count = Subsidies_HDI_SI.groupby(['HDI_group','Class'], as_
index=False).count()
# group subsidy intensity by calculating average (mean) per HDI group an
d per subsidy Type (Class)
# using count to see how many countries we have data for, for each subsi
dy type
```

In [22]:
```
Unknown_grouped = Unknown.groupby(['Country', 'Class'], as_index=False).
sum()
# now we prepare 'unknown amount' data, where we find clear evidence of
 a subsidy type existing but no amount,
# by shortening dataframe to keep only necessary columns

Unknown_grouped = Unknown_grouped[['Country', 'Class']]
# as unknown amounts are entered at subtype basis we need to group by su
sbidy type (Class)

Unknown_grouped['Subsidies Constant 2018 USD'] = ('unknown')
# create a new columns with the same name as the column in original dat
 that contains subsidy amounts and
# fill with the string 'unknown'
# this is important to not get confused with zeros (0) entered in origin
al data
# i.e. data that was clearly found as zero and entered as such

Subsidies_2018 = pd.concat([Subsidies_2018_Types, Unknown_grouped], igno
re_index=True)
# concat means to add the unknown dataframe underneath the original Subs
idies_2018_Types.
# all column headers have to be the same
```

In [23]:
```
Subsidies2009 = Subsidies2009[['RegionName','Country','Category','Class'
,'Type','Subsidy_2009_USD']]
Subsidies_all = pd.merge(left=Subsidies2009, right=Subsidies_2018, how=
'left')
# make Subsidies2009 baseline dataframe and merge 2018 estimates onto le
ft side - merge based on Country and Class
```

In [24]:
```
# before estimating all Types using Landed Value, we take fuel data out
# to use fuel consumption information instead of landed value data to es
timate fuel separately

Subsidies_2018_HDI_con = pd.merge(left = Subsidies_2018_HDI,
                                  right = Fuel_consumption, how='left')
# we use dataframe already merged with HDI groups as basis and add (merg
e) fuel consumption information

Subsidies_2018_Fuel = Subsidies_2018_HDI_con.loc[(Subsidies_2018_HDI_con
["Class"] == 'B7'), :]
# we slice the dataframe by takeing out only fuel subsidy type (Class =
 B7)

Subsidies_2018_Fuel = Subsidies_2018_Fuel[['Country', 'Class', 'Subsidie
s Constant 2018 USD',
                                           'HDI_group','fuel_aver_tonnes
_2009_2018']]

# reduce dataframe to only necessary columns

Subsidies_2018_Fuel['Subsidy per tonne'] = Subsidies_2018_Fuel['Subsidie
s Constant 2018 USD'] /Subsidies_2018_Fuel['fuel_aver_tonnes_2009_2018']
# similar to subsidy intensity by using subsidies/LV, we use Fuel subsid
y/fuel consumed i.e. subsidy per tonne of fuel


Fuel_con_average = Subsidies_2018_Fuel.groupby(['HDI_group'], as_index=F
alse).mean()
# calculate average subsidy per tonne of fuel per HDI group (high and lo
w)

Fuel_con_average_only = Fuel_con_average[['HDI_group','Subsidy per tonn
e']]
# reduce dataframe cto only necessary columns
```

In [25]:
```
Fuel_only = Subsidies_all.loc[(Subsidies_all["Class"] == 'B7'), :]
# now we take the entire subsidy dataframe (including unknown amounts, a
nd data gaps) and slice into only fuel
```

In [26]:
```
Fuel_HDI = pd.merge(left = Fuel_only, right = Countries_HDI, how = 'lef
t')
# we add HDI grop information to fuel only
```

In [27]:
```
Fuel_subsidies_con = pd.merge(left=Fuel_HDI, right= Fuel_consumption, ho
w='left')
# add fuel consumption info to fuel only dataframe
Fuel_subsidies_con = Fuel_subsidies_con.loc[:, ['RegionName','Country',
'Category', 'Class','Type','Subsidy_2009_USD',
                                                'Subsidies Constant 2018
USD', 'HDI_group',
                                                'fuel_aver_tonnes_2009_2
018']]
# reduce dataframe to only necessary columns

Fuel_subsidies = pd.merge(left = Fuel_subsidies_con, right = Fuel_con_av
erage_only, how = 'left')
# add average subsidy per tonne of fuel per HDI group

Fuel_subsidies['estimated_fuel'] = Fuel_subsidies['fuel_aver_tonnes_2009
_2018'] * Fuel_subsidies['Subsidy per tonne']
# calculate 'estimated fuel' by multiplying average subsidy per tonne of
fuel with fuel consumption per country
```

In [28]:
```
def is_valid(val):
    if isinstance(val, str) or val in [None, np.nan]:
        return False
    return True

# function describes when there are no data in the cell,
# this does not include zero (0) and does not include 'unknown'
```

In [29]:
```
def fuel(row):
    if row['Subsidies Constant 2018 USD'] == 'unknown':
            val = row['estimated_fuel']

    elif not is_valid(row['Subsidies Constant 2018 USD']) and not row['S
ubsidy_2009_USD'] == 0:
            val = row['estimated_fuel']

    elif not row['Subsidies Constant 2018 USD'] == np.nan:
            val = row['Subsidies Constant 2018 USD']
    else:
        val = row['estimated_fuel']
    return val

# function to use where estimated fuel data is used to fill gaps and whe
re not

Fuel_subsidies['estimated_fuel_2018'] = Fuel_subsidies.apply(fuel, axis=
1)
# apply function and fill data in a new column
# the function determines which data is used,
# if an amount (this inlcudes '0') exists in 'subsidies constant 2018 US
D' then the amount is used;
# if 'unknown' data point, then use 'estimated fuel';
# if no data entered but a data point exists in 2009 then use 'estimated
fuel'
```

In [30]:
```
# To include a column that describes 'Observed' (reported) vs 'Modeled'
 data point see function below
def observed_fuel(row):
    if not is_valid(row['estimated_fuel_2018']):
        return 'NA'

    elif row['Subsidies Constant 2018 USD'] == row['estimated_fuel_2018'
]:
        return 'Reported'

    else:
        return 'Modeled'

Fuel_subsidies['Actual_data vs modeled_estimates'] = Fuel_subsidies.appl
y(observed_fuel, axis=1)
# create new column that indicates observed vs modeled data
```

In [31]:
```
# continue with all subsidy types to be merged with fuel data at the end
```

In [32]:
```
HDI_LV_all = pd.merge(left=HDI, right=EEZ_LV, how='left')
# merge Landed Value data (EEZ)
HDI_all_LV = pd.merge(left=HDI_LV_all, right=Fleet_LV, how='left')
# merge with Landed Value data Fleet
```

In [33]:
```
Subsidies_all_LV = pd.merge(left=Subsidies_all, right=HDI_all_LV, how='l
eft')
# merge all countries subsidy data by type with HDI and LV data by count
ry
```

In [34]:
```
Subsidies_all_short = Subsidies_all_LV.loc[:, ['RegionName','Country','C
ategory','Class','Type',
                                        'Subsidy_2009_USD','Subsidies Co
nstant 2018 USD',
                                        'HDI_group','FleetLV_USD_2014',
'EEZLV_2014_USD']]
# reduce dataframe to only necessary columns
```

In [35]:
```
Subsidies_all_SI = pd.merge(left=Subsidies_all_short, right=Subsidies_SI
, how='left')
# merge all subsidies dataframe (as basis) with Subsidiy Intensity avera
ges per HDI group
```

In [36]:
```
Subsidies_all_estimates = pd.merge(left=Subsidies_all_SI, right=Type_Fle
et_EEZ, how='left')
# add dataframe that describes which subsidy type will be used fleet and
for which EEZ landed value dada
```

In [37]:
```
Subsidies_all_estimates['Estimated_2018_USD'] = np.where(Subsidies_all_e
stimates['LV_Type']=='EEZ',
                                                          Subsidies_all_e
stimates['EEZLV_2014_USD']*
                                                          Subsidies_all_e
stimates['Subsidy_intensity'],
                                                          Subsidies_all_e
stimates['FleetLV_USD_2014']*
                                                          Subsidies_all_e
stimates['Subsidy_intensity'])
# if 'LV_Type' is equal to 'EEZ' then calculate ['Subsidy_intensity'] *
 ['EEZLV_2014_USD']
# if 'LV_Type is equal to 'Fleet' then calculate ['Subsidy_intensity'] *
['FleetLV_USD_2014']
```

In [38]:
```
Subsidies_model2018 = Subsidies_all_estimates.loc[:, ['RegionName','Coun
try','Category','Class',
                                                       'Type','Subsidy_20
09_USD','Subsidies Constant 2018 USD',
                                                       'Estimated_2018_US
D']]
# reduce dataframe to only necessary columns
```

In [39]:
```
def merge(row):
    if row['Class'] == 'A3'or row['Class'] == 'B6':
        val = row['Subsidies Constant 2018 USD']

    elif row['Class'] == 'A1':
        if is_valid(row['Subsidies Constant 2018 USD']):
            val = row['Subsidies Constant 2018 USD']
        else:
            val = row['Estimated_2018_USD']

    elif row['Class'] in ['A2','B1', 'B2', 'B3', 'B4', 'B5', 'B7', 'C1',
'C2', 'C3']:
        if row['Subsidies Constant 2018 USD'] == 'unknown':
            val = row['Estimated_2018_USD']

        elif is_valid(row['Subsidies Constant 2018 USD']):
            val = row['Subsidies Constant 2018 USD']

        elif not is_valid(row['Subsidies Constant 2018 USD']) and not ro
w['Subsidy_2009_USD'] == 0:
            val = row['Estimated_2018_USD']

        else:
            val = row['Subsidies Constant 2018 USD']
    else:
        val = row['Estimated_2018_USD']
    return val

# the function 'merge' uses an if elif and else argument to define which
data are being used in the new column
# for Classes A3 and B6, which are MPA and Access, and which are already
modeled seperately, we always use
# the original amount copied directly from 'subsidies constant 2018 USD'
# for all A1, management. we copy data from 'subsidies constant 2018 US
D' if it contains a value (is_valid) if
# it does not the gao is filled with 'estimated_2018_USD' amount
# for all othe classes we use 'subsidies constant 2018 USD' amount if ex
isting
# if not we check if a existing in 2009 data
# if yes we fill gap with 'estimated_2018_USD' if not 2009 data exist we
fill with 0
```

In [40]:
```
Subsidies_model2018['Estimated_2018_all'] = Subsidies_model2018.apply(me
rge, axis=1)
# we create a new column in the data frame which applies the 'merge' fun
ction
```

In [41]:
```
def observed_modeled(row):
    if not is_valid(row['Estimated_2018_all']):
        return 'NA'

    elif row['Class'] == 'A3'or row['Class'] == 'B6':
        return 'Modeled'

    elif row['Subsidies Constant 2018 USD'] == row['Estimated_2018_all'
]:
        return 'Reported'

    else:
        return 'Modeled'
# to know which data in the newly created column is observed vs modeled
 data we apply the function 'observed_modeled

Subsidies_model2018['Actual_data vs modeled_estimates'] = Subsidies_mode
l2018.apply(observed_modeled, axis=1)
# we create a new column and apply the function modeled-observed to indi
cate which amount in 'Estimated_2018_all'
# stems from original observed (reported) data versus modeled
```

In [42]:
```
# as fuel is still included in above model, we now take all fuel data ou
t and replace with fuel data modeled/observed
# from the model part using fuel consumption information
```

In [43]:
```
Subsidies_nofuel_2018 = Subsidies_model2018.loc[(Subsidies_model2018["Cl
ass"] != 'B7'), :]
# take out all ['Class'] == 'B7' rows, which are fuel subsidy data point
s
```

In [44]:
```
Fuel_subsidies_merge = Fuel_subsidies.loc[:, ['RegionName','Country','Cl
ass','Category','Type', 'Subsidy_2009_USD',
                                              'Subsidies Constant 2018 U
SD','estimated_fuel','estimated_fuel_2018',
                                              'Actual_data vs modeled_es
timates']]
# reduce dataframe to only necessary columns

Fuel_subsidies_merge.columns = ['RegionName','Country','Class','Categor
y','Type','Subsidy_2009_USD',

                       'Subsidies Constant 2018 USD', 'Estimated_2018_US
D','Estimated_2018_all',
                                'Actual_data vs modeled_estimates']

# re-name columns to match Subsidies_nofuel_2018 dataframe to prepare fo
r joining it with fuel subsidies

# Fuel_subsidies_merge = pd.merge(left = Fuel_subsidies_merge, right = S
ubsidies2009, how = 'left')
# merge data with 2009 subsidies

Fuel_subsidies_merge_rearrange = Fuel_subsidies_merge[['RegionName', 'Co
untry', 'Category', 'Class',
                                                       'Type','Subsidy_2
009_USD', 'Subsidies Constant 2018 USD',
                                                       'Estimated_2018_U
SD', 'Estimated_2018_all',
                                                       'Actual_data vs m
odeled_estimates']]
# re arrange columns
```

In [45]:
```
Subsidies_2018_final = pd.concat([Subsidies_nofuel_2018, Fuel_subsidies_
merge_rearrange], ignore_index=True)
# add fuel datrame to no_fuel dataframe
Subsidies_2018_final.columns
```

Out[45]:
```
Index(['RegionName', 'Country', 'Category', 'Class', 'Type',
       'Subsidy_2009_USD', 'Subsidies Constant 2018 USD', 'Estimated_20
18_USD',
       'Estimated_2018_all', 'Actual_data vs modeled_estimates'],
      dtype='object')
```

In [46]:
```
Subsidies_update_2018 = Subsidies_2018_final.loc[:, ['RegionName', 'Coun
try', 'Class', 'Category', 'Type',
                                                        'Estimated_2018_al
l', 'Actual_data vs modeled_estimates']]
# reduce dataframe to only necessary columns

Subsidies_update_2018.columns = ['Region Name', 'Country', 'Class', 'Cat
egory', 'Type', 'Constant 2018 USD',
                                 'Data Type']
# rename columns

Subsidies_update_2018 = Subsidies_update_2018.sort_values(by = ['Region
 Name','Country', 'Class'], ascending = True)
# Sort data by region and by country

Subsidies_update_2018['Category'] = Subsidies_update_2018['Category'].re
place('Bad','Capacity-enhancing')
Subsidies_update_2018['Category'] = Subsidies_update_2018['Category'].re
place('Good','Beneficial')
Subsidies_update_2018['Category'] = Subsidies_update_2018['Category'].re
place('Ugly','Ambiguous')
# replace terminology of Categories

Subsidies_update_2018 = Subsidies_update_2018.fillna(0)
# fill all blanks with zeros


Subsidies_update_2018['Data Type New'] = np.where(Subsidies_update_2018[
'Data Type'] == 'Reported', 'Reported',
                                                  (np.where((Subsidies_u
pdate_2018['Data Type'] == 'Modeled') &
                                                  (Subsidies_update_2018
['Constant 2018 USD'] == 0),
                                                  'not found evidence of
subsidy','Modeled')))

# if Subsidies_update_2018['Data Type'] == 'modeled' and Subsidies_updat
e_2018['Constant 2018 USD'] == 0 then
# 'not found evidence of subsidy'

Subsidies_update_2018 = Subsidies_update_2018.loc[:, ['Region Name', 'Co
untry', 'Class', 'Category',
                                                  'Type', 'Constant 2018
USD', 'Data Type New']]

Subsidies_update_2018.columns = ['Region Name', 'Country', 'Class', 'Cat
egory', 'Type', 'Constant 2018 USD',
                                 'Data Type']
```

In [47]:
```
Subsidies_final_country_data = pd.merge(left=Subsidies_update_2018, righ
t = Countrydata, how = 'left')
# add group information for easy pivot tables if exported to excel
Subsidies_final_country_data.columns
```

Out[47]:
```
Index(['Region Name', 'Country', 'Class', 'Category', 'Type',
       'Constant 2018 USD', 'Data Type', 'RegionName', 'Subregion', 'CL
on',
       'CLat', 'EU member', 'HDI_2017', 'HDI_group', 'Rank', 'ACP',
       'Least TRUE', 'Commonwealth', 'SIDS', 'UN_Developed'],
      dtype='object')
```

In [48]:
```
Subsidies_final_developed = Subsidies_final_country_data.loc[:, ['Region
Name', 'Country', 'Class', 'Category', 'Type',
                                                                 'Consta
nt 2018 USD', 'Data Type','UN_Developed']]

Subsidies_final_developed['UN_Developed'] = Subsidies_final_developed['U
N_Developed'].replace(True,'Developed')
Subsidies_final_developed['UN_Developed'] = Subsidies_final_developed['U
N_Developed'].replace(False,'Developing')

# prepare data to be grouped by developed vs developing based on UN cate
gorization
```

In [49]:
```
Countries_HDI_short = Countries_HDI.loc[:, ['Country','HDI_group']]

Subsidies_final_HDI = pd.merge(left=Subsidies_update_2018, right = Count
ries_HDI_short, how = 'left')

Subsidies_final_HDI_count = Subsidies_final_HDI.groupby(['Data Type','HD
I_group','Class'], as_index = False).count()
# prepare data to be grouped by High and Low HDI (based on UN) and to se
e how many countries fall in each HDI group
```

In [50]:
```
# Take raw 2019 data that include sources to prep for Appendix,
# needs to be merged with model output at the end
# so all sources are indicated in an extra column for all countries and
 all Types

Subsidies2019_sources = Subsidies2019[['Country','Class', 'Source']]
# shorten dataframe to only necessary columns

Subsidies2019_sources = Subsidies2019_sources.drop_duplicates(keep='firs
t',inplace=False)
# drop duplicates to prepare for groupby

Subsidies2019_sources['Source'] = Subsidies2019_sources['Source'].str.re
place(r'[^\x00-\x7F]+', '')
# delete all Chinese characters
```

In [51]:
```
Sources_2019_grouped = Subsidies2019_sources.groupby(['Country', 'Class'
])['Source'].apply(list).reset_index()
#  concatenate source strings, this adds the source names from each Subt
ypes and groups by Type

dupes = []  #  store duplicate index in a list
for index, row in Sources_2019_grouped.iterrows():
    source = row['Source']
    if len(source) != len(set(source)):
       dupes.append(index)
print(f'dupes: {dupes}')
#  check for duplicates to make sure all are deleted, if still duplicate
s present dupes:[] would show them below.
```

```
dupes: []
```

In [52]:
```
Subsidies_final_sources = pd.merge(left = Subsidies_update_2018, right =
Sources_2019_grouped, how ='left')
Subsidies_final_sources = Subsidies_final_sources.loc[:,['Region Name',
'Country', 'Type','Constant 2018 USD', 'Data Type', 'Source']]
Subsidies_final_sources.columns = ['Region Name', 'Country', 'Type','Sub
sidies amount (USD in 2018)', 'Data Type', 'Source of information']
Subsidies_final_sources = Subsidies_final_sources.fillna('')
# add all source names prepared above to final subsidies dataframe to pr
epare for Appendix
```
